# GABA Concentration in the Left Ventral Premotor Cortex Associates With Sensory Hyper-Responsiveness in Autism Spectrum Disorders Without Intellectual Disability

**DOI:** 10.3389/fnins.2020.00482

**Published:** 2020-05-19

**Authors:** Yumi Umesawa, Takeshi Atsumi, Mrinmoy Chakrabarty, Reiko Fukatsu, Masakazu Ide

**Affiliations:** ^1^Department of Rehabilitation for Brain Functions, Research Institute of National Rehabilitation Center for Persons with Disabilities, Saitama, Japan; ^2^Department of Medical Physiology, Faculty of Medicine, Kyorin University, Tokyo, Japan; ^3^Department of Social Sciences and Humanities, Indraprastha Institute of Information Technology (IIIT-D), New Delhi, India

**Keywords:** autism spectrum disorder, gamma-aminobutyric acid, sensory hyper-responsiveness, magnetic resonance spectroscopy, ventral premotor cortex

## Abstract

Individuals with autism spectrum disorder (ASD) often exhibit abnormal processing of sensory inputs from multiple modalities and higher-order cognitive/behavioral response to those inputs. Several lines of evidence suggest that altered γ-aminobutyric acid (GABA), the main inhibitory neurotransmitter in the brain, is a central characteristic of the neurophysiology of ASD. The relationship between GABA in particular brain regions and atypical sensory processing in ASD is poorly understood. We therefore employed ^1^H magnetic resonance spectroscopy (^1^H-MRS) to examine whether GABA levels in brain regions critical to higher-order motor and/or multiple sensory functions were associated with abnormal sensory responses in ASD. We evaluated atypical sensory processing with a clinically-validated assessment tool. Furthermore, we measured GABA levels in four regions: one each in the primary visual cortex, the left sensorimotor cortex, the left supplementary motor area (SMA), and the left ventral premotor cortex (vPMC). The latter two regions are thought to be involved in executing and coordinating cognitive and behavioral functions in response to multisensory inputs. We found severer sensory hyper-responsiveness in ASD relative to control participants. We also found reduced GABA concentrations in the left SMA but no differences in other regions of interest between ASD and control participants. A correlation analysis revealed a negative association between left vPMC GABA and the severity of sensory hyper-responsiveness across all participants, and the independent ASD group. These findings suggest that reduced inhibitory neurotransmission (reduced GABA) in a higher-order motor area, which modulates motor commands and integrates multiple sensory modalities, may underlie sensory hyper-responsiveness in ASD.

## Introduction

Individuals with autism spectrum disorder (ASD) often exhibit sensory abnormalities [for more, see Marco et al. (2011)]. Sensory hyper- and hypo-responsiveness are frequently observed in autistic individuals, although this is not part of the core definition of autism (APA, 2013). There is individual variation in the sensory modalities that are most disrupted in individuals with autism, and the sensory abnormalities could be seen in all sensory domains (Kientz and Dunn, 1997; Tomchek and Dunn, 2007; Lane et al., 2014). Findings from clinical contexts have revealed abnormal sensory processing in autism, not just in the sensitivity to sensory inputs, but also in later cognitive/behavioral reactivity, including passive avoiding and/or seeking external stimuli (Lane et al., 2014; Damiano-Goodwin et al., 2018; Schulz and Stevenson, 2019). Sensory processing involves registration and modulation of sensory information, as well as an internal organization of afferent inputs (Humphry, 2002). Indeed, sensory hyper-responsiveness is a key feature included in the restricted interests and repetitive behaviors central to an ASD diagnosis (APA, 2013), and some studies have further demonstrated that sensory stimuli detection sensitivity was insufficient to describe the severity of sensory hyperresponsiveness in autism (Ide et al., 2019; Schulz and Stevenson, 2019). Altered sensory processing therefore, may occur in the stream of information processing involving higher-order cognitive processing (Thye et al., 2018).

The molecular biology of autism has revealed that altered γ-aminobutyric acid (GABA)-mediated signaling within some brain circuits, may explain the sensory abnormalities seen in ASD (Rubenstein and Merzenich, 2003; Cellot and Cherubini, 2014; Braat and Kooy, 2015; Foss-Feig et al., 2017; Robertson and Baron-Cohen, 2017). Altered inhibitory GABAergic transmission may lead to an abnormal excitatory/inhibitory balance in the brain, which can alter neural signaling and information processing, as well as responding behavior (Foss-Feig et al., 2017). Recent *in vivo* studies have also revealed reduced GABA concentrations across multiple cortical areas of the autistic brain (Harada et al., 2011; Gaetz et al., 2014; Rojas et al., 2014; Puts et al., 2017; Sapey-Triomphe et al., 2019). Collectively, these findings indicate that altered GABAergic signaling may be related to the abnormal daily sensory experience of individuals with autism.

The aim of the present study was to examine the relationship between subjectively evaluated atypical sensory processing and GABA concentrations in primary sensory and motor areas and cortical regions involved in higher-order cognitive and behavioral functions. While higher-order motor related areas have been implicated in action responses and multimodal cognitive processes (Rizzolatti et al., 2014), whether GABA concentrations in those areas correlate with abnormal sensory processing in ASD remains unknown. To assess this, we measured GABA concentrations in multiple brain regions using ^1^H magnetic resonance spectroscopy (^1^H-MRS) in the present study.

We analyzed two major higher-order motor regions-the ventral premotor cortex (vPMC) and the supplementary motor area (SMA), which have been demonstrated to have tight neural connections with sensorimotor cortex in humans and primates (Luppino et al., 1993; Yeo et al., 2011). Previous studies have suggested that the vPMC is involved in multiple sensory processing (Iacoboni and Dapretto, 2006; Bekrater-Bodmann et al., 2011; Ide et al., 2020), especially for response modulation or inhibition to sensory signals when a change of the reaction patterns is needed (Buch et al., 2010). As the execution of motor sequences and imitation of actions involved in higher-order motor areas further lateralizes to the left hemisphere (Hlustík et al., 2002; Vingerhoets et al., 2013; Reader and Holmes, 2018), we hypothesized that weakened inhibition in the left vPMC would associate with atypical sensory processing observed in ASD. The SMA is known to be involved in voluntary motor execution, motor planning, and coordinated body movements (Roland et al., 1980; Tanji et al., 1988; Sumner and Husain, 2008) rather than functions in the sensory domains. We further assessed two additional regions [the primary visual cortex (V1) (Robertson et al., 2016) and the sensorimotor cortex (SMC) (Puts et al., 2017)] in which GABA levels and perceptual performance may be related and abnormal in ASD.

## Materials and Methods

### Participants

Seventeen adolescent and adult participants with ASD (12 males) and 18 typically developing (TD) participants (11 males) were recruited. Demographic data for both groups are summarized in Table 1. Individuals with a clinical diagnosis of ASD were recruited from parent groups of children with developmental disorders and the Department of Child Psychiatry at the National Rehabilitation Center for Persons with Disabilities. We recruited all the participants by random sampling, regardless of their genetic background and diagnosis of abnormal sensory processing. It should also be noted that we have no information regarding any motor disabilities in each individual. None of the participants recruited in this study were excluded from the analysis. To assess the validity of diagnostic group differences, we used the Japanese version of the Autism Quotient (AQ) scale (Baron-Cohen et al., 2001; Wakabayashi et al., 2004), in which higher scores indicate stronger autistic traits. None of the TD participants had AQ scores above the threshold (cut-off: 33) and a two-tailed *t* test revealed significantly higher AQ scores in ASD participants than in TD controls (*t*_33_ = 5.162, *p* < 0.01, Cohen’s *d* = 1.75). One female ASD participant (age 23), who did not receive a clinical diagnosis, was included in the ASD group because of her AQ score of 37, which exceeded the diagnostic threshold. We further used the Wechsler Adult Intelligence Scale-Third Edition (WAIS-III) to assess participant Intelligence Quotients (IQs). No participants had full-scale IQs below 75. All participants and their parents gave written informed consent for study participation after all study procedures were fully explained. The present study was approved by the Ethics committee of the National Rehabilitation Center for Persons with Disabilities. The present experiment adhered to institutional safety procedures for human brain imaging. Note that the participants and their ^1^H-MRS data of the left SMC and SMA were partially overlapped with those employed in Umesawa et al. (2020); 14 ASD (three females) and 11 TD (five females) participants.

**TABLE 1:** Demographic information and differences between groups.

	ASD group	TD group
Sex (M:F)	12:5 (*N* = 17)	11:7 (*N* = 18)
Age, mean years (range)	21.5 ± 3.2	22.7 ± 6.0
LQ, mean (range)	68.9 ± 36.9	82.1 ± 33.3
AQ, mean (range)**	32.6 ± 8.1	20.1 ± 6.2
VIQ, mean (range)	111.8 ± 16.0	115.5 ± 12.1
PIQ, mean (range)	105.4 ± 17.8	112.4 ± 13.5
FIQ, mean (range)	109.4 ± 14.2	115.6 ± 11.3

**p < 0.01. M, male; F, female; ASD, autism spectrum disorder; TD, typically developing; LQ, laterality quotient; AQ, Autism spectrum Quotient; VIQ, verbal intelligence quotient; PIQ, performance intelligence quotient; FIQ, full-scale intelligence quotient. The AQ score was evaluated by the Autism spectrum Quotient (AQ) scale. The LQ score was assessed using the Edinburgh Handedness Inventory (Oldfield, 1971). The intellectual quotients (IQs) were assessed by the Wechsler Adult Intelligence-Third Edition (WAIS-III). Asterisk indicates significant difference between groups found by two-tailed *t* test.

### Adolescent/Adult Sensory Profile

We evaluated individual sensory responsiveness using the Japanese version of the Adolescent/Adult Sensory Profile (AASP) (Brown et al., 2001), which originated from Dunn’s model of sensory processing disorders (Dunn, 1997) and is based on Ayres’ theory of sensory integration (Ayres, 1979). The AASP is broadly accepted for the characterization of altered sensation in individuals with ASD and is a subjective questionnaire which consists of 60 items classified into four subscales (normal range): low registration (23–38), sensation seeking (30–47), sensory sensitivity (25–42), and sensation avoiding (25–41). Low registration reflects how easily an individual misses sensory information, while sensation seeking indicates a tendency to seek out sensory stimulation. Sensory sensitivity indicates a heightened awareness of sensory stimuli and sensory avoiding reflects a tendency to withdraw from strong sensory input. The first two scales indicate the severity of sensory hypo-responsiveness and the others represent hyper-responsiveness (Dunn, 2001).

### MR Acquisition

We acquired magnetic resonance imaging (MRI) data on a 3T Siemens Skyra scanner (Siemens, Erlangen, Germany) with a 64-channel head coil. We ran two sessions with a sequence designed to obtain anatomical images and two sequences for ^1^H-MRS within a day (i.e., each participant underwent four sessions total). First, we obtained a high-resolution *T1*-weighted anatomical image using a magnetization-prepared rapid acquisition by gradient echo sequence [number of slices = 224, slice thickness = 1 mm, repetition time (TR) = 2300 ms, echo time (TE) = 2.98 ms, flip angle = 9°] to set regions of interest (ROIs) with a voxel size of 20 mm × 20 mm × 20 mm (Nakai and Okanoya, 2016). Based on this anatomical image, we manually determined different ROIs (see section “Regions of Interest (ROI)”) across multiple sessions.

### Regions of Interest (ROI)

**FIGURE 1:**
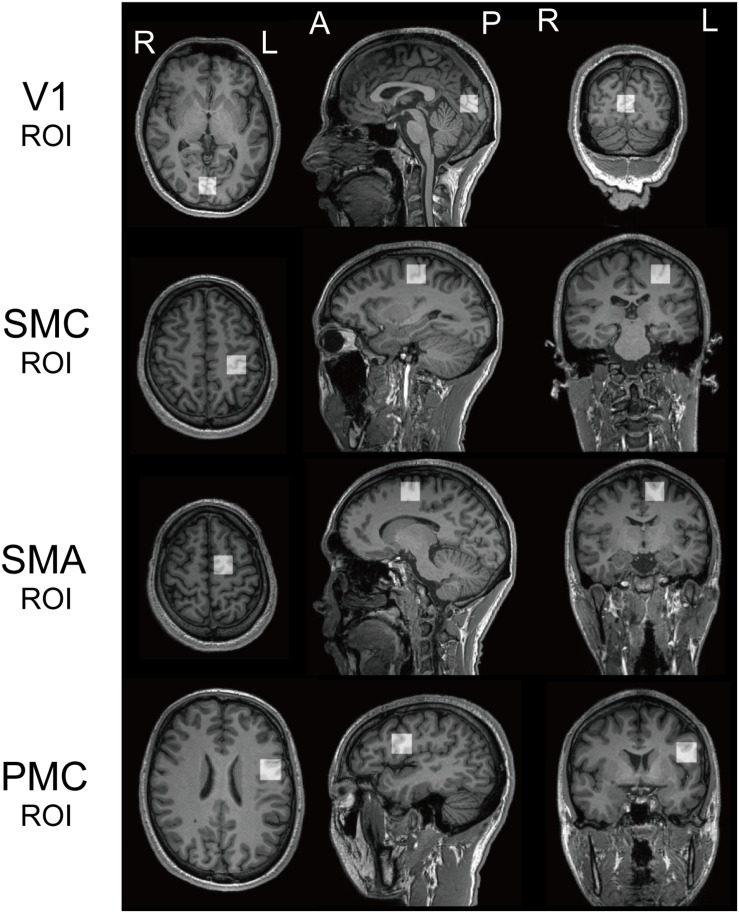
Regions of interest. Regions of interest (ROI) for ^1^H magnetic resonance spectroscopy for both populations. V1, the bilateral primary visual cortices; SMC, the left sensory motor cortex; SMA, the left supplementary motor area; PMC, the left ventral premotor cortex.

Recent studies have found that specific perceptual functions, are associated with an atypical role for GABA in several ASD brain regions. We set two ROIs, the bilateral V1 and the left SMC based on those previous knowledges, in addition to the left SMA and vPMC (referred to as PMC: Figure 1).

Typically developing individuals with higher GABA concentrations in the visual cortex exhibited increased suppression of visual perception, but this association was absent in ASD participants (Robertson et al., 2016). Autistic children with higher GABA levels in the sensorimotor cortex had lower sensitivity to vibrotactile input amplitude after adaptation to it (Puts et al., 2017). While neurotypical children with higher GABA levels in that region exhibited greater sensitivity to the frequency of a given stimuli, children with autism didn’t exhibit this (Puts et al., 2017). Another study examined associations between subjective individual difficulties in sensory processing, a psychophysical index, and somatosensory cortex GABA levels (Sapey-Triomphe et al., 2019). This study reported higher GABA levels and higher frequencies of atypical tactile experiences (as per a self-reported questionnaire) in individuals with ASD.

The anatomical definitions of ROIs were as follows; the V1 ROI was midline of the occipital cortex (Muthukumaraswamy et al., 2012). The SMC ROI included the “hand-knob” of the left central sulcus (Yousry et al., 1997). The SMA ROI was the superior and medial part of Brodmann area (BA) 6, with its inferior face anterior to the cingulate sulcus and extending to the dorsal premotor cortex. The PMC ROI included the lower and lateral parts of BA6, with its inferior face anterior to the lateral sulcus [mainly including the ventral PMC (vPMC)]. We used a MEGA-PRESS sequence for GABA-edited MRS (Mescher et al., 1998) to quantify GABA in each ROI (TR = 2000 ms; TE = 70 ms; 128 averages; 20 mm × 20 mm × 20 mm). We used LCModel (Provencher, 2001) to quantify resultant spectra and calculated a ratio of GABA+ (reflecting GABA+ co-edited macro-molecules) to N-acetyl aspartate acid (NAA) to quantify the GABA concentration in each ROI (Harada et al., 2011; Gaetz et al., 2014).

## Results

### AASP Scores

**FIGURE 2:**
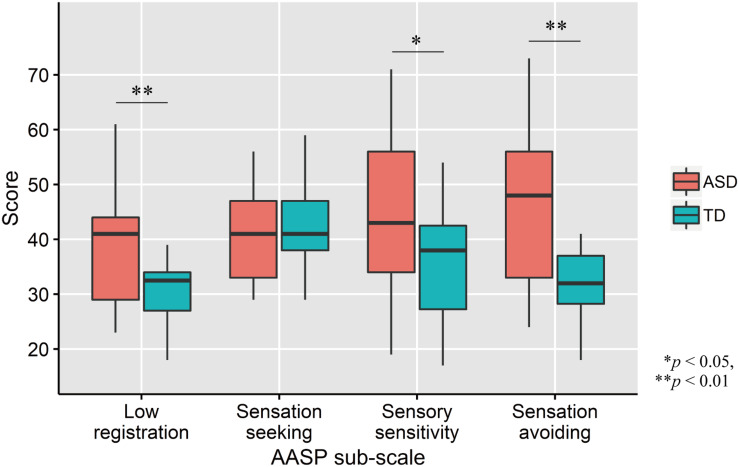
Distribution of AASP scores in each group. The upper and lower boundaries of the standard boxplots represent the 25th and 75th percentiles. The horizontal line across the box marks the median of the distribution. The ends of vertical lines below and above the box represent the minimum and maximum values, respectively. Asterisks represent significant difference by two-tailed *t* test.

Comparisons of AASP scores by two-tailed *t* test revealed that ASD participants had significantly greater low registration (*t*_33_ = 3.01, *p* = 0.005, *d* = 1.02), sensory sensitivity (*t*_33_ = 2.03, *p* = 0.05, *d* = 0.69), and sensation avoiding (*t*_33_ = 3.09, *p* = 0.004, *d* = 1.04) scores, but not sensation seeking (*t*_33_ = −0.77, *p* = 0.45, *d* = −0.26; Figure 2) scores, than TD controls.

### GABA+ Concentrations

**FIGURE 3:**
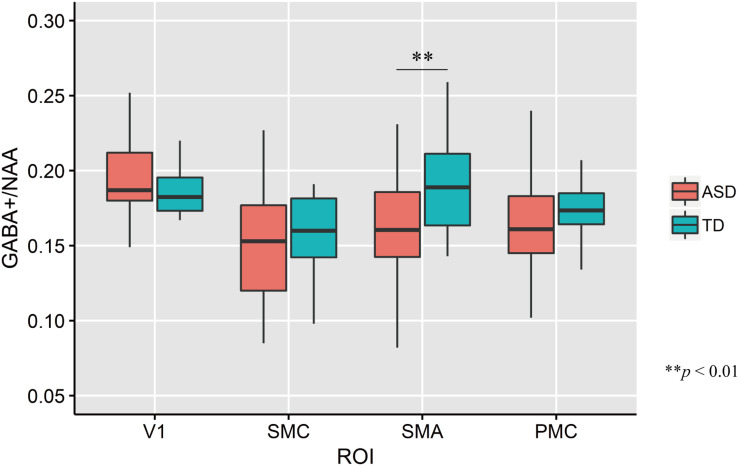
Distribution of GABA+/NAA ratio of every ROIs in each group. The upper and lower boundaries of the standard boxplots represent the 25th and 75th percentiles. The horizontal line across the box marks the median of the distribution. The ends of vertical lines below and above the box represent the minimum and maximum values, respectively. Asterisks represent significant difference by two-tailed *t* test. V1, the bilateral primary visual cortices; SMC, the left sensory motor cortex; SMA, the left supplementary motor area; PMC, the left ventral premotor cortex.

The mean GABA+ concentrations across four ROIs in each group are shown in Figure 3. The mean GABA+ /NAA ratio in the left SMA of ASD participants was significantly lower than that of TD controls (two-tailed *t* test: *t*_33_ = −2.74, *p* = 0.01, *d* = −0.93). No other regions had significant group-wise differences (V1: *t*_33_ = 1.35, *p* = 0.19, *d* = 0.46; PMC: *t*_33_ = −0.65, *p* = 0.52, *d* = −0.22; SMC: *t*_33_ = −0.64, *p* = 0.52, *d* = −0.22).

### Correlation Analyses

**FIGURE 4:**
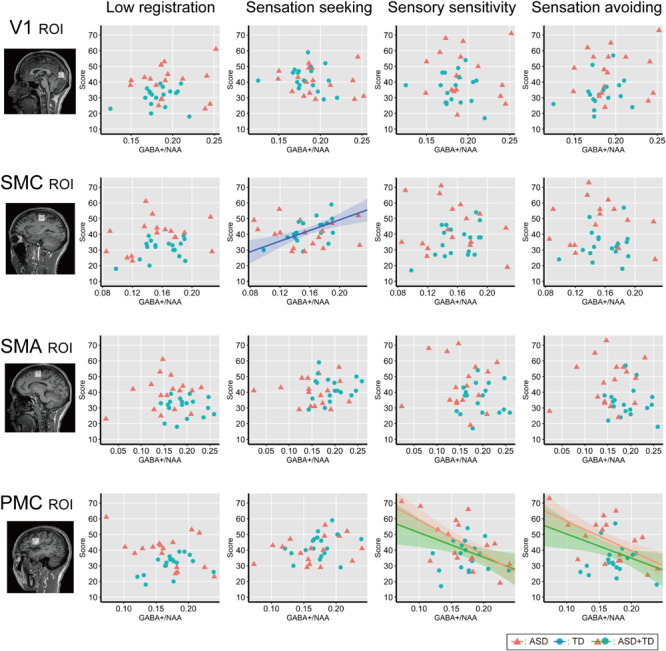
Correlation analysis between individual AASP and GABA+ level. Individual sub-scale scores are plotted against individual GABA+ /NAA ratio in each ROI. Red triangles indicate ASD individuals and blue circles indicate TD individuals. Shaded bands represent 95% confidence intervals across individuals for each group (green: all participants, red: ASD group, blue: TD group). V1, the bilateral primary visual cortices; SMC, the left sensory motor cortex; SMA, the left supplementary motor area; PMC, the left ventral premotor cortex.

Figure 4 shows associations between individual GABA+ /NAA ratios and each AASP subscale score across the ROIs. Correlation analyses across all participants (*N* = 35) revealed negative correlations between GABA+ /NAA ratios and sensory sensitivity (Pearson’s correlation coefficient *r* = −0.43, *p* = 0.01, 95% confidence interval (CI) = [−0.67, −0.11]) and sensation avoiding scores (*r* = −0.41, *p* = 0.013, CI = [−0.66, −0.09]) in the PMC. Some associations were significant in ASD participants, including those for sensory sensitivity (*r* = −0.63, *p* = 0.007, CI = [−0.80, −0.38]) and sensation avoiding (*r* = −0.59, *p* = 0.014, CI = [−0.77, −0.31]), but not in the TD group (*p* > 0.7 for both). Furthermore, there was a significant positive correlation between GABA+ /NAA ratio in the left SMC and sensation seeking in the TD group (*r* = 0.56, *p* = 0.015, CI = [0.28, 0.76]), but not in the ASD group (*p* > 0.6). No other subscales were significantly associated with GABA+ levels in either all participants or independently in either of the two groups.

## Discussion

The present study assessed whether GABA+ concentrations in specific brain areas were associated with different domains of abnormal sensory experiences in individuals with ASD. An analysis of sensory processing patterns, as assessed by a self-report questionnaire, revealed that participants with ASD had increased low registration, sensory sensitivity, and sensation avoiding subscale scores. Although we found a difference in GABA+ relative to NAA concentrations only in the left SMA between the ASD and TD groups, the other ROIs, the bilateral V1, the left SMC, and the left vPMC did not differ in this measure. Correlation analyses demonstrated that individuals with lower GABA+ levels in the left vPMC had increased sensory hyper-responsiveness (i.e., higher scores in the sensory sensitivity and sensation avoiding subscales of the AASP). This trend was obvious in ASD but not in TD participants. Recent studies in transgenic mice with deletions of autism-related genes have further revealed that reduced GABA-mediated inhibitory signals can induce hyper-responsiveness to sensory stimuli (Orefice et al., 2016, 2019; He et al., 2017). Our finding suggest that reduced inhibitory neurotransmission, caused by reduced GABA in the higher-order motor areas that modulate motor commands and integrate sensory information across multiple modalities, is related to increased sensory hyperresponsiveness in ASD.

Atypical sensory processing in people with ASD would involve behavioral patterns in extraordinary response to sensory inputs and not just restricted in sensitivity (e.g., low threshold of perception: Ide et al., 2019; Schulz and Stevenson, 2019). Two major higher-order motor-related areas, the SMA and vPMC have connections with sensorimotor cortices in human and non-human primates (Luppino et al., 1993; Yeo et al., 2011). Previous studies have suggested that the vPMC is involved in multiple sensory processing, especially for response modulation or inhibition to sensory signals when a change of the reaction patterns is needed (Buch et al., 2010). The vPMC is involved in low-level sensory encoding and motor functions, such as goal-directed behavior in response to multiple sensory information (Rizzolatti et al., 2002). For instance, in hand grasping, this area is critical in using visual information to for appropriately shaping hands (Rizzolatti et al., 2002; Davare et al., 2008; Prabhu et al., 2009). Furthermore, inhibition of M1 activity by the vPMC is critical for rapid behavioral modulation based on action plan changes (Buch et al., 2010). Neurons in the primate vPMC respond to multiple sensory inputs, especially to tactile stimuli and partially to visual (Gentilucci et al., 1988) and auditory stimuli (Graziano et al., 1999). A human functional MRI study demonstrated that the left vPMC activated during tactile orientation judgment (Zhang et al., 2005). Previous work has shown that the secondary motor (M2) area in mice, which is functionally homologous to the human PMC, has synchronized neural activity with the primary somatosensory area and is crucial for tactile texture discrimination (Manita et al., 2015). Given those and the present result, atypical neural modulation of earlier cortical regions by the vPMC may lead to sensory and motor processing dysfunction.

Previous accounts of highly cognitive domains, such as temporal processing of stimulus, in ASD may also implicate links between the left vPMC and sensory hyper-responsiveness given reduced inhibitory cortical neuron activity (Poole et al., 2017; Ide et al., 2019). Our previous study elucidated that individuals with ASD who showed high resolution of tactile stimulus temporal order tended to have severer sensory hyper-responsiveness (Yaguchi et al., 2020). Functional MRI studies have demonstrated that multiple cortical regions are involved in temporal order judgment of multisensory stimuli, including the left vPMC, which likely plays a key role (Takahashi et al., 2013; Binder, 2015; Miyazaki et al., 2016; Ide et al., 2020). The vPMC is also involved in bodily awareness, which may derive from the integration of visual and somatosensory information (Bekrater-Bodmann et al., 2011; Brozzoli et al., 2012). The vPMC is also involved in sensation and decision-making during auditory discrimination (Lemus et al., 2009). Considering the findings presented here, the vPMC may additional be involved in the awareness of multiple sensory stimuli, leading to later action responses to these inputs. Because of its integrative function, reduced inhibitory neurotransmission in the left vPMC in ASD may induce increased reactivity across multiple stages of sensory processing in ASD (Dunn, 1997; Schulz and Stevenson, 2019).

The data presented here are somewhat inconsistent with a previous study which reported greater GABA levels in the sensorimotor cortex and a higher frequency of subjective atypical tactile experiences in individuals with ASD (Sapey-Triomphe et al., 2019). In this previous work, however, the researchers evaluated sensory hypersensitivity and hyposensitivity using the same index and by extracting only tactile domain features. The difference between our own work and this prior study may be due to a focus on modality-dependent predictability of ordinary stimuli (Sapey-Triomphe et al., 2019) and the GABA in the corresponding primary sensory region. In the present study, AASP was used, which differentiates between hypersensitivity and hyposensitivity using Dunn’s model (Dunn, 1997). Additionally, given the multisensory processing role of the vPMC, this region is likely more closely related to domain-general atypical sensory hyper-responsiveness, as reported previously in functional associations between GABA and psychophysical measurements in autism (Robertson et al., 2016; Puts et al., 2017).

Despite early reports of reduced GABA across brain areas, we found significant reductions only in the left SMA and no difference in other regions. Most children with ASD have co-morbid developmental coordination disorder, which reflects dysfunction in coordinated body movements (Green et al., 2009). The SMA is thought to be essential for coordinated body movements (Roland et al., 1980; Tanji et al., 1988; Sumner and Husain, 2008). In agreement with our recent report, the present study’s finding indicates that reduced GABA+ in the SMA may reflect complicated motor disability in ASD (Umesawa et al., 2020). Prior work has found that GABA concentrations in V1 did not differ between adults with ASD and controls, but rather were associated with functional measures that characterized that population (Robertson et al., 2016). Additional studies have reported reduced GABA in the sensorimotor cortex in autistic children (Gaetz et al., 2014; Puts et al., 2017), though only one study has reported this in autistic adults (Sapey-Triomphe et al., 2019). At present, little is known about GABA concentrations in frontal areas, including higher-order motor regions, in individuals with ASD. One study demonstrated significant reductions in frontal lobe GABA in children with autism compared to controls and no changes in striatal GABA levels (Harada et al., 2011). Another study of adults with ASD revealed no differences in GABA concentrations in either the medial prefrontal cortex or the striatum (Horder et al., 2018). Critically, GABA concentration may also change with age (Clement et al., 1987; McQuail et al., 2015; Rowland et al., 2016). Our findings in adolescents and adults reveal that increased variation of cerebral GABA concentration across the participants by age might reduce the clear between-group difference.

In the present study, GABA+ levels in the left SMC in TD participants were positively correlated with their sensation seeking index scores, which measures one’s preference for behaviors being proximal to stimuli to create a sensation (Brown et al., 2001; Dunn, 2001). Previous work in an autism-unrelated mouse strain demonstrated that GABAergic parvalbumin neurons in the primary motor cortex are essential for the inhibition of sensory-triggered motor reaction behaviors (Estebanez et al., 2017). The present study suggests that individual variation in sensorimotor GABA+ levels modulates subjective impulsivity and associated responses to external stimuli, but not in individuals with ASD. Previous work in autistic adults has reported that sensation seeking in ASD individuals differed from the other three scales (Crane et al., 2009). Our sample did not replicate sensation seeking abnormalities in participants with ASD, but this cognitive/behavioral aspects of ASD may reflect another potential association between the neurobiological and pathognomonic traits of autism.

We should note that the findings of our study from a small cohort has limitations to be extended to the larger population. We had no information regarding genetic backgrounds of each individual. Some autism-related genes have been considered to relate to GABAergic inhibition (Orefice et al., 2016, 2019; He et al., 2017). We also did not screen for any motor disabilities of the participants. Thus, whether these uncontrolled factors had any influence on our present results is unknown.

To date, a number of studies in ASD patients have examined modality-dependent atypical sensory processing and respective neural correlations. Although individual variability in sensory modality abnormalities and later behavioral response in ASD are well known, associations between clinically-validated sensory assessments and brain metabolites are less well understood. The present study is the first to comprehensively analyze the relationship between GABA+ levels in multiple brain regions and multiple aspects of sensory processing deficits in ASD. As discussed above, the left vPMC may be involved in the processing of multiple sensory information, though its specific function which accounts for sensory processing disorder in autism remains unknown. Future work should examine whether a specific cognitive capacity which the left vPMC is involved in, such as temporal processing of stimuli (Takahashi et al., 2013; Binder, 2015; Miyazaki et al., 2016; Ide et al., 2020), bodily-awareness (Bekrater-Bodmann et al., 2011; Brozzoli et al., 2012), and decision-making (Lemus et al., 2009), mediates the association between GABA and atypical sensory processing. Furthermore, whether there is an altered role for GABA in the vPMC in individuals with autism should be evaluated by whole-brain functional and anatomical connectivity (Ameis et al., 2016; Yahata et al., 2016). Findings from those studies may allow us to evaluate the possibility of GABA levels in the left vPMC as a significant biomarker and therapeutic target for autistic sensory processing disorder.

## Data Availability Statement

The datasets generated for this study are available on request to the corresponding author.

## Ethics Statement

The studies involving human participants were reviewed and approved by Ethics committee of the National Rehabilitation Center for Persons with Disabilities. Written informed consent to participate in this study was provided by the participants’ legal guardian/next of kin.

## Author Contributions

YU, TA, MC, and MI conceived the study. YU, TA, and MI conducted the experiments. YU and TA analyzed the data. All authors interpreted the results read the manuscript, gave relevant inputs, and approved the final version of the same. YU, TA, and MI wrote the manuscript.

## Conflict of Interest

The authors declare that the research was conducted in the absence of any commercial or financial relationships that could be construed as a potential conflict of interest.
